# Right-to-left shunt detection using contrast-enhanced transcranial Doppler: A comparison of provocation maneuvers between coughing and a modified Valsalva maneuver

**DOI:** 10.1371/journal.pone.0175049

**Published:** 2017-04-06

**Authors:** Si-Bo Wang, Xiao-Cong Wang, Yan Ma, Kang-Ding Liu, Ying-Qi Xing

**Affiliations:** 1 Neuroscience Center, Department of Neurology, The First Hospital of Jilin University, Changchun, China; 2 Department of Echocardiography, Cardiovascular Center, The First Hospital of Jilin University, Changchun, China; National Natural Science Foundation of China, CHINA

## Abstract

Contrast-enhanced transcranial Doppler (c-TCD) has been used to detect right-to-left shunts (RLS) because it is highly sensitive and cost-effective. The use of provocation maneuvers, such as physiologic maneuvers (e.g., coughing) and the Valsalva maneuver (VM) to transiently increase right atrial pressure and induce RLS increases the sensitivity of RLS detection. In this study, we sought to determine whether coughing is as effective as the VM in aiding the detection of RLS. We evaluated 162 subjects for RLS, using c-TCD under three different conditions: (i) resting state, (ii) coughing, and (iii) modified VM (m-VM), which involved blowing into a tube connected to a sphygmomanometer at 40 mmHg for 10 s. The positive rate of RLS detection with the m-VM was significantly higher than that with coughing. In addition, a difference between the two maneuvers was observed in terms of the degree of RLS seen. The m-VM should be widely used to detect RLS, because it is reliable, standardized, and cost-effective.

## Introduction

Right-to-left shunt (RLS) has recently been reported as a risk factor for cryptogenic stroke, migraine, and decompression sickness [1–4]. A previously published study also found that provoked RLS (i.e., RLS that occurs only after a provocation maneuver) is associated with vertebrobasilar lesions in patients with ischemic stroke [5]. Contrast-enhanced transcranial Doppler (c-TCD) is a clinically applicable and reproducible method for detecting RLS, including intracardiac and extracardiac RLS.

Provocation maneuvers aim to raise right atrial pressure and increase the sensitivity of RLS detection [6, 7]. These maneuvers are therefore recommended when detecting RLS and in routine clinical practice. Several different provocation maneuvers are used for this purpose, including physiologic maneuvers, the conventional Valsalva maneuver (c-VM), and the standard or modified Valsalva maneuver (m-VM) [8–10]. A simple cough has been used as a physiologic maneuver to provoke RLS [8, 9]. Unfortunately, the most recent International Consensus Meeting did not provide explicit instructions regarding the standard procedure for the VM [7]. In a recent study, authors reported that m-VM provoked a much higher positive rate of RLS detection than c-VM (hold expiration and release to transiently increase right arterial pressure), with higher detection rates observed in c-TCD [11].

These different provocation maneuvers are used in RLS detection tests, including contrast-enhanced transthoracic echocardiography (c-TTE), contrast-enhanced transesophageal echocardiography (c-TEE), and c-TCD [6, 8]. The relative accuracy and detection rates of these tests are the source of much controversy and discussion. However, most authors use the VM as a provocation maneuver in c-TCD tests, while coughing and the VM are used in c-TTE and c-TEE studies to increase the sensitivity of RLS detection.

With this in mind, we sought to determine whether coughing is as effective as the m-VM as a provocation maneuver for RLS detection during c-TCD.

## Methods

### Participants

**Fig 1:**
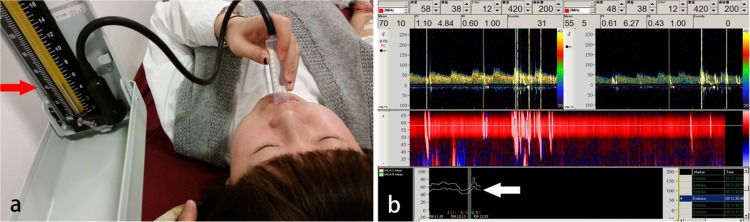
Quality control of the modified and conventional Valsalva maneuvers. (a) The red arrow shows 40 mmHg on the sphygmomanometer. (b) The white arrow shows the Doppler flow velocity curve (down first and then up) (adapted from Guo *et al*. with permission [11]).

The study design was approved by the Ethics Committee of the First Hospital of Jilin University. We enrolled 162 patients (44 men) with a mean age of 39.04 ± 10.26 years (range, 16–65 years), who were referred to the Department of Neurology of our hospital between October 2015 and April 2016, due to a strong suspicion of RLS. Written informed consent was obtained from all enrolled participants and the methods were performed in accordance with approved guidelines. The individual in Fig 1 has given written informed consent to publish these case details. Of the patients enrolled, 158 had headaches, three dizziness, and one unilateral upper limb numbness. Among patients with headaches, 135 were diagnosed with migraine and eight with tension-type headaches based on the International Headache Society Criteria [12]. Patients with severe arterial stenosis, an insufficient temporal window, inadequate cubital venous access, and those unable to perform the m-VM because of severe heart or lung disease were excluded.

### Contrast-enhanced transcranial Doppler

All subjects underwent c-TCD and c-TTE simultaneously, which were performed by two independent operators. A baseline TCD examination was performed with a TCD detector (EMS-9A, Delica, China). We used a hand-held 2-MHz probe at the right middle cerebral artery (MCA) with participants lying comfortably in the left lateral position. c-TTE was performed using the GE Vivid E9 platform and M5S transducer (Horten, Norway). Four-chamber views were obtained to optimize visualization of the interatrial septum. An 18-gauge catheter was placed in the right antecubital vein. The medium was prepared by hand by mixing 9 mL of saline, 1 mL of air, and a drop of the participant’s own blood [13]. The medium was rapidly mixed back-and-forth with two 10-mL syringes connected by a three-way stopcock 30 times to create microbubbles (MBs).

The procedure was performed using three different conditions: (i) RS, (ii) the simple cough maneuver, where the patient vigorously coughed for 10 s immediately after the medium injection [10], and (iii) m-VM. The cough maneuver was determined to be adequate if a leftward shift of the atrial septum was present on c-TTE imaging. For the m-VM, participants were coached to blow into the connecting tube of a sphygmomanometer at 40 mm Hg for a 10-s period, starting 5 s after the initiation of medium injection [7, 11]. After the RS test, patients were randomly assigned to either cough or to undergo the m-VM test first. Measurements were performed twice with the simple cough method and twice with the m-VM; RLS was rated based on the highest number of MBs detected in the 20-s duration of each c-TCD run [7]. There was an interval of at least 5 minutes from the last observed MB between tests. MBs were defined as a typical chirping sound and visually by the spike-like appearance in the frequency spectrum and M-mode. Two blinded ultrasound technicians assessed the prevalence and extent of RLS.

**Fig 2:**
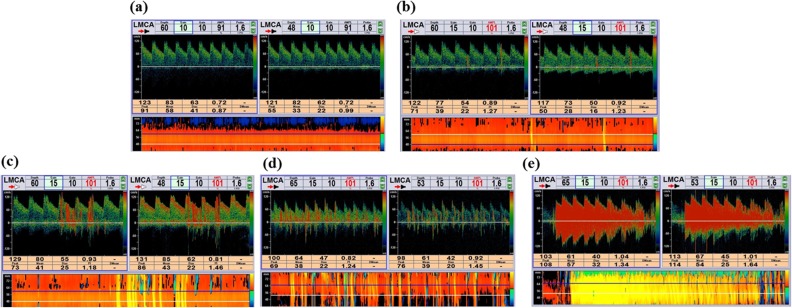
The five-level right-to-left shunt categorization according to microbubble (MB) count in the contrast-enhanced transcranial Doppler spectrum using unilateral middle cerebral artery monitoring. Grade 0 = negative (a); Grade I = 1 ≤ MBs ≤ 10 (b); Grade II = 10 < MBs ≤ 25 (c); Grade III = >25 MBs and no curtain (d); Grade IV = curtain (where a single bubble cannot be identified) (e).

Several different categorization systems exist for RLS [3, 7, 14, 15]. Based on standards reported by Jauss *et al*., Wessler *et al*., and Xing *et al*. [7, 15, 16], a five-level categorization system was used in this study based on the appearance of MBs in the TCD spectrum using unilateral MCA monitoring as follows: Grade 0, negative; Grade I, 1 ≤ MBs ≤ 10; Grade II, 10 < MBs ≤ 25; Grade III, > 25 MBs and no curtain; and Grade IV, curtain where a single bubble cannot be identified (Fig 2).

### Statistics

Statistical analyses were performed using SPSS 17.0 (Chicago, IL, USA). The0020difference between the detection rates of the different maneuvers was analyzed using the chi-square test. Data are presented as numbers (%). McNemar’s test was used to compare the positive rates of RLS detection between the different tests maneuver/c-TCD combinations. Bowker’s test was used to compare the extent of RLS between coughing and the m-VM. The level of significance was set at a P-value < 0.05.

## Results

### Positive rate of RLS detection

The positive rates of RLS detected with c-TCD were 56.2% (91 of 162), 82.1% (133 of 162), and 89.5% (145 of 162) for RS, coughing, and the m-VM, respectively. The differences between these detection rates were statistically significant (chi square, 54.30; P < 0.001) (Table 1). Using either the m-VM or coughing method increased the positive rate of shunt detected by c-TCD, compared with using the RS method (Tables 2 and 3). Furthermore, the positive detection rate with the m-VM was significantly higher than that with coughing (89.5% vs. 82.1%, P = 0.017) (Table 4).

**Table 1:** Positive rates of right-to-left shunt (RLS) detection in the resting state (RS), cough, and modified Valsalva maneuver (m-VM) conditions by contrast-enhanced transcranial Doppler.

		RLS		Total
		Negative	Positive	
State	RS (n/%)	71 (43.8)	91 (56.2)	162 (100.0)
	Cough (n/%)	29 (17.9)	133 (82.1)	162 (100.0)
	m-VM (n/%)	17 (10.5)	145 (89.5)	162 (100.0)

Pearson’s chi-square = 54.304, P = 0.00.

**Table 2:** Cross-tabulation of resting state (RS) and cough for right-to-left shunt detection.

		Cough	
		Positive	Negative
RS	Positive	85	6
	Negative	48	23

P = 0.00.

**Table 3:** Cross-tabulation of resting state (RS) and the modified Valsalva maneuver (m-VM) for right-to-left shunt detection.

		m-VM	
		Positive	Negative
RS	Positive	85	6
	Negative	60	11

P = 0.00.

**Table 4:** Cross-tabulation of cough and the modified Valsalva maneuver (m-VM) for right-to-left shunt detection.

		m-VM	
		Positive	Negative
Cough	Positive	128	5
	Negative	17	12

P = 0.017.

### Severity of RLS

**Fig 3:**
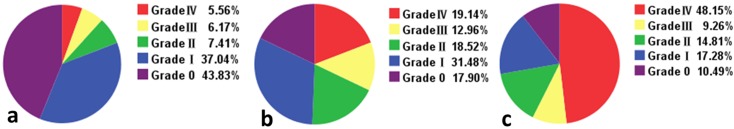
Positive detection rates of the different right-to left shunt degrees in (a) the resting state (RS), (b) cough, (c) the modified Valsalva manoeuver (m-VM) conditions.

Overall, the degree of RLS during c-TCD using the RS method were 37.04% (60 of 162) grade I, 7.41% (12 of 162) grade II, 6.17% (10 of 162) grade III, and 5.56% (9 of 162) grade IV. Using the simple cough method, 31.48% (51 of 162) were grade I, 18.52% (30 of 162) grade II, 12.96% (21 of 162) grade III, and 19.14% (31 of 162) grade IV. For the m-VM, 17.28% (28 of 162) were grade I, 14.81% (24 of 162) grade II, 9.26% (15 of 162) grade III, and 48.15% (78 of 162) grade IV (Fig 3).

Based on the five-level categorization system used in this study, the degree of RLS detected using the simple cough method was consistent with that detected using the m-VM in 55 of 162 patients: 22 were grade IV, two grade III, seven grade II, 12 grade I, and 12 negative. The majority of disagreements (86/107) were within the high-degree m-VM/low-degree simple cough section of the matrix rather than in the high-degree cough/low-degree m-VM section of the matrix. There was a significant difference between the simple cough method and m-VM in terms of the degree of RLS detected (P < 0.001) (Table 5).

**Table 5:** Cross-tabulation of cough and the modified Valsalva maneuver (m-VM) for determining the degree of right-to-left shunt.

		m-VM					Total
		Grade IV	Grade III	Grade II	Grade I	Grade 0	
Cough	Grade IV	22	3	5	1	0	31
	Grade III	17	2	0	1	1	21
	Grade II	14	3	7	6	0	30
	Grade I	21	5	9	12	4	51
	Grade 0	4	2	3	8	12	29
Total		78	15	24	28	17	162

P = 0.00.

## Discussion

In this study, we evaluated whether using the simple cough method, as a provocation maneuver, is as effective as the m-VM for RLS detection. We found that the m-VM yielded a higher detection rate for RLS compared to the simple cough maneuver, and also lead to higher degrees of RLS being detected. Altogether, these data suggest that the m-VM is a more effective provocation maneuver than coughing to detect RLS.

RLS has been associated with migraine, cryptogenic stroke, and decompression sickness [1–4]. Accurate RLS evaluation via c-TCD is needed in this patient population [17]. c-TCD in combination with a provocation maneuver has been widely used for RLS detection, because it is highly sensitive, non-invasive, and cost-effective [10]. In some patients, MBs can only be detected after using a provocation maneuver, while in others, MBs can be detected during normal respiration. Provocation maneuvers that increase right atrial pressure have been shown to enhance the probability of RLS detection; previously tested maneuvers include the simple cough method and VM [9, 18, 19]. In a previous study, investigators found that RLS could be provoked in 34.2% of cryptogenic stroke patients with a patent foramen ovale (PFO); MBs were detected only after provocation maneuvers by c-TCD [5]. Thus, an effective provocation maneuver during RLS detection is clinically important.

It is well known that several different provocation maneuvers are used to provoke RLS, including physiologic maneuvers, as well as the c-VM and m-VM [9, 10]. The reliability and overall clinical utility of these different provocation maneuvers are a source of much controversy. In 1993, Stoddard *et al*. reported that coughing was superior to the VM in diagnosing patients with intracardiac RLS using c-TEE [20]. The VM was performed after deep inspiration and maintained for 5 s. The simple cough maneuver was performed by the patient coughing three–five times rapidly after the opacification of the right atrium with agitated saline solution. However, this observation was not confirmed by c-TCD. A possible explanation for these controversial results may be that patients under oropharyngeal anesthesia can perform coughing maneuvers more easily than those undergoing the VM during c-TEE. Zanette *et al*. compared the incidence rates of RLS using coughing or the VM during c-TCD in 38 participants with PFO. In their study, the VM was performed by holding expiration and releasing the strain. Furthermore, the authors evaluated the VM by defining a successful attempt as a mean flow velocity reduction of at least 25%. The results showed that VM lead to the detection of slightly more positive cases than the simple cough maneuver, with a higher number of MBs detected in each patient [21]. The results of the present study are also in accordance with the results of Zanette *et al*. We demonstrated that the m-VM yielded a higher positive detection rate than coughing during c-TCD (89.5% vs. 82.1%, P = 0.017). Furthermore, we enrolled 162 symptomatic participants, which is a significantly higher than the number of patients enrolled in the Zanette *et al*. study, allowing us to gain a more accurate clinical picture. In clinical practice, many patients have difficulty comprehending the instructions that must be followed when performing the c-VM. The strength of the c-VM was measured using peak flow velocity (Fig 1). The m-VM, which required patients to blow at 40 mm Hg for 10 s, was much easier for patients to master and had other advantages, such as the possibility of quantitatively assessing the patient’s ability to perform the maneuver. The ability of patients to perform provocation maneuvers effectively is very important in RLS detection. The increased sensitivity provided by performing effective provocation maneuvers may help reveal RLS in patients who were diagnosed with cryptogenic stroke, migraine, and other diseases associated with RLS.

RLS comprises intracardiac and extracardiac RLS [22]. Intracardiac RLS usually occurs through a PFO [23]. Stone *et al*. found that patients with a large degree of interatrial RLS across a PFO, as determined by c-TEE, had a significantly greater risk for subsequent adverse neurologic events than those with a lesser degree of RLS [24]. With emerging observational studies and clinical trials targeting PFOs, patients with cryptogenic stroke or severe migraine and intracardiac RLS through a PFO are increasingly considered for transcatheter closure [17, 25]. When considering the treatment of patients with cryptogenic stroke and interatrial RLS across a PFO, it is clinically important to analyze the severity of RLS and identify the possibility of ‘guilty’ or ‘innocent’ RLS. Possible treatment modalities for the prevent of recurrent stroke events among cryptogenic stroke patients with RLS though a PFO include medical treatment with warfarin or antiplatelet agents, percutaneous PFO closure, and surgical PFO closure [26]. To date, whether medical therapy or surgical PFO closure is the most beneficial in preventing PFO-RLS-related strokes remains controversial. In 2012, Furlan *et al*. reported that closure with a device did not offer a greater benefit than medical therapy alone in preventing recurrent stroke or transient ischemic attack (TIA) in patients with cryptogenic stroke or TIA with PFO [27]. Stone *et al*. reported that patients with larger shunts (more than 20 MBs of contrast passing between the atria within three cardiac cycles) should receive such treatment, while it is recommended that patients with smaller shunts be followed-up conservatively [24]. A systematic review of three randomized controlled trials provided insufficient support that PFO closure is preferable to medical therapy for the secondary prevention of cryptogenic stroke in patients with PFO [28]. Furthermore, recommendations from the American Heart Association/American Stroke Association exist for the prevention of recurrent stroke in a variety of specific circumstances, including intracardiac RLS with PFO [29]. In view of these treatment recommendations, an effective provocation maneuver for quantifying the degree of RLS seems necessary to guide therapeutic decision-making for patients with RLS.

Although the findings of this study were novel and clinically important, there were also several limitations. We did not perform comparisons of the c-TEE findings in our study. However, the main aim of this study was not to determine the cause of RLS, but rather to compare the ability of two different provocation maneuvers to facilitate RLS detection in the same patient. There are several different methods of performing the VM. A standard procedure of the VM is not available, and the m-VM provoked a much higher positive rate of RLS detection than c-VM [11]. Considering m-VM as an effective provocation method, we sought to determine whether coughing is as effective as m-VM instead of c-VM. Most of the authors used either coughing or the VM as the provocative maneuver during TTE and TEE, while VM alone tended to be used for c-TCD. In this study, we used c-TCD to evaluate whether coughing is as effective as m-VM. Future studies are warranted to reassess and verify these results using c-TTE and c-TEE to establish that the superiority of the m-VM is true for all available modalities.

## Conclusion

The m-VM is a more effective RLS provocation maneuver than coughing in detecting RLS. The simple cough maneuver is relatively “non-standard” compared to with the m-VM, in that it is not easy to quantitatively assess each performance. The m-VM is therefore preferable for assessing RLS, and should be used in clinical practice.

## Supporting information

S1 File The ethics committee form. (PDF)

S2 File Written informed consent of the participant. (PDF)

S3 File Certificate of English Editing. (PDF)

S1 Video Quality control of the cough maneuver using contrast-enhanced transthoracic echocardiography. (7Z)

## References

[1] Anzola GP: Clinical impact of patent foramen ovale diagnosis with transcranial Doppler. European journal of ultrasound: official journal of the European Federation of Societies for Ultrasound in Medicine and Biology 2002; 16(1–2): 11–20. 10.1016/s0929-8266(02)00043-5 12470846

[2] Xu WH, Xing YQ, Yan ZR, Jiang JD, Gao S: Cardiac right-to-left shunt subtypes in Chinese patients with cryptogenic strokes: a multicenter case-control study. European journal of neurology 2014; 21(3): 525–528. doi: 10.1111/ene.12351 24444328 10.1111/ene.12351

[3] Yang Y, Guo ZN, Wu J, Jin H, Wang X, Xu J, et al Prevalence and extent of right-to-left shunt in migraine: a survey of 217 Chinese patients. European journal of neurology 2012; 19(10): 1367–1372. doi: 10.1111/j.1468-1331.2012.03793.x 22747847 10.1111/j.1468-1331.2012.03793.x

[4] Gempp E, Blatteau JE, Stephant E, Louge P: Relation between right-to-left shunts and spinal cord decompression sickness in divers. International journal of sports medicine 2009; 30(2): 150–153. doi: 10.1055/s-2008-1038844 18773377 10.1055/s-2008-1038844

[5] Kim BJ, Kim NY, Kang DW, Kim JS, Kwon SU: Provoked right-to-left shunt in patent foramen ovale associates with ischemic stroke in posterior circulation. Stroke; a journal of cerebral circulation 2014; 45(12): 3707–3710. 10.1161/STROKEAHA.114.007453 25358696

[6] Jauss M, Kaps M, Keberle M, Haberbosch W, Dorndorf W: A comparison of transesophageal echocardiography and transcranial Doppler sonography with contrast medium for detection of patent foramen ovale. Stroke; a journal of cerebral circulation 1994; 25(6): 1265–1267. 10.1161/01.str.25.6.1265 7911265

[7] Jauss M, Zanette E: Detection of right-to-left shunt with ultrasound contrast agent and transcranial Doppler sonography. Cerebrovascular diseases (Basel, Switzerland) 2000, 10(6):490–496. 10.1159/000016119 11070388

[8] Silvestry FE, Cohen MS, Armsby LB, Burkule NJ, Fleishman CE, Hijazi ZM, et al Guidelines for the Echocardiographic Assessment of Atrial Septal Defect and Patent Foramen Ovale: From the American Society of Echocardiography and Society for Cardiac Angiography and Interventions. Journal of the American Society of Echocardiography: official publication of the American Society of Echocardiography 2015; 28(8): 910–958. 26239900 10.1016/j.echo.2015.05.015

[9] Droste DW, Kriete JU, Stypmann J, Castrucci M, Wichter T, Tietje R, et al Contrast transcranial Doppler ultrasound in the detection of right-to-left shunts: comparison of different procedures and different contrast agents. Stroke; a journal of cerebral circulation 1999; 30(9): 1827–1832. 10.1161/01.str.30.9.1827 10471431

[10] Sastry S, MacNab A, Daly K, Ray S, McCollum C. Transcranial Doppler detection of venous-to-arterial circulation shunts: criteria for patent foramen ovale. Journal of clinical ultrasound: JCU 2009; 37(5): 276–280. doi: 10.1002/jcu.20564 19353576 10.1002/jcu.20564

[11] Guo YZ, Gao YS, Guo ZN, Niu PP, Yang Y, Xing YQ. Comparison of Different Methods of Valsalva Maneuver for Right-to-left Shunt Detection by Contrast-Enhanced Transcranial Doppler. Ultrasound in medicine & biology 2016; 42(5): 1124–1129. 26928233 10.1016/j.ultrasmedbio.2015.12.020

[12] The International Classification of Headache Disorders, 3rd edition (beta version). Cephalalgia: an international journal of headache 2013; 33(9): 629–808. 23771276 10.1177/0333102413485658

[13] Hao N, Liu K, Guo ZN, Wu X, Yang Y, Xing Y. Comparison of two contrast agents for right-to-left shunt diagnosis with contrast-enhanced transcranial Doppler. Ultrasound in medicine & biology 2014; 40(9): 2317–2320. 25023098 10.1016/j.ultrasmedbio.2014.03.011

[14] Lao AY, Sharma VK, Tsivgoulis G, Frey JL, Malkoff MD, Navarro JC, et al Detection of right-to-left shunts: comparison between the International Consensus and Spencer Logarithmic Scale criteria. Journal of neuroimaging: official journal of the American Society of Neuroimaging 2008; 18(4): 402–406. 18333839 10.1111/j.1552-6569.2007.00218.x

[15] Wessler BS, Kent DM, Thaler DE, Ruthazer R, Lutz JS, et al The RoPE Score and Right-to-Left Shunt Severity by Transcranial Doppler in the CODICIA Study. Cerebrovascular diseases (Basel, Switzerland) 2015; 40(1–2): 52–58. 10.1159/000430998 PMC4523222 26184495

[16] Xing YQ, Guo YZ, Gao YS, Guo ZN, Niu PP, Yang Y. Effectiveness and Safety of Transcatheter Patent Foramen Ovale Closure for Migraine (EASTFORM) Trial. Scientific reports 2016; 6: 39081 doi: 10.1038/srep39081 27966652 10.1038/srep39081 PMC5155423

[17] Mojadidi MK, Roberts SC, Winoker JS, Romero J, Goodman-Meza D, Gevorgyan R, et al Accuracy of transcranial Doppler for the diagnosis of intracardiac right-to-left shunt: a bivariate meta-analysis of prospective studies. JACC Cardiovascular imaging 2014; 7(3): 236–250. doi: 10.1016/j.jcmg.2013.12.011 24560213 10.1016/j.jcmg.2013.12.011

[18] Aparci M, Guney Senol M, Yalcin M, Tansel Kendirli M, Isilak Z. Effective Valsalva maneuvering during TCCD and unrevealed etiology of RLS. Acta neurologica Scandinavica 2016; 133(4): 313–314. doi: 10.1111/ane.12485 26935909 10.1111/ane.12485

[19] Pfleger S, Konstantin Haase K, Stark S, Latsch A, Simonis B, Scherhag A, et al Haemodynamic quantification of different provocation manoeuvres by simultaneous measurement of right and left atrial pressure: implications for the echocardiographic detection of persistent foramen ovale. European journal of echocardiography: the journal of the Working Group on Echocardiography of the European Society of Cardiology 2001; 2(2): 88–93. 10.1053/euje.2000.0052 11882433

[20] Stoddard MF, Keedy DL, Dawkins PR. The cough test is superior to the Valsalva maneuver in the delineation of right-to-left shunting through a patent foramen ovale during contrast transesophageal echocardiography. American heart journal 1993; 125(1): 185–189. 8417516 10.1016/0002-8703(93)90073-i

[21] Zanette EM, Mancini G, De Castro S, Solaro M, Cartoni D, Chiarotti F. Patent foramen ovale and transcranial Doppler. Comparison of different procedures. Stroke; a journal of cerebral circulation 1996; 27(12): 2251–2255. 10.1161/01.str.27.12.2251 8969789

[22] Nedeltchev K, Mattle HP. Contrast-enhanced transcranial Doppler ultrasound for diagnosis of patent foramen ovale. Frontiers of neurology and neuroscience 2006; 21: 206–215. doi: 10.1159/000092432 17290139 10.1159/000092432

[23] Anzola GP, Magoni M, Guindani M, Rozzini L, Dalla Volta G. Potential source of cerebral embolism in migraine with aura: a transcranial Doppler study. Neurology 1999; 52(8): 1622–1625. 10331688 10.1212/wnl.52.8.1622

[24] Stone DA, Godard J, Corretti MC, Kittner SJ, Sample C, Price TR, et al Patent foramen ovale: association between the degree of shunt by contrast transesophageal echocardiography and the risk of future ischemic neurologic events. American heart journal 1996; 131(1): 158–161. 8554004 10.1016/s0002-8703(96)90065-4

[25] Rengifo-Moreno P, Palacios IF, Junpaparp P, Witzke CF, Morris DL, Romero-Corral A. Patent foramen ovale transcatheter closure vs. medical therapy on recurrent vascular events: a systematic review and meta-analysis of randomized controlled trials. European heart journal 2013; 34(43): 3342–3352. doi: 10.1093/eurheartj/eht285 23847132 10.1093/eurheartj/eht285

[26] Homma S, Sacco RL. Patent foramen ovale and stroke. Circulation 2005; 112(7): 1063–1072. doi: 10.1161/CIRCULATIONAHA.104.524371 16103257 10.1161/CIRCULATIONAHA.104.524371

[27] Furlan AJ, Reisman M, Massaro J, Mauri L, Adams H, Albers GW, et al Closure or medical therapy for cryptogenic stroke with patent foramen ovale. The New England journal of medicine 2012; 366(11): 991–999. doi: 10.1056/NEJMoa1009639 22417252 10.1056/NEJMoa1009639

[28] Spencer FA, Lopes LC, Kennedy SA, Guyatt G. Systematic review of percutaneous closure versus medical therapy in patients with cryptogenic stroke and patent foramen ovale. BMJ open 2014; 4(3): e004282 doi: 10.1136/bmjopen-2013-004282 24607561 10.1136/bmjopen-2013-004282 PMC3948581

[29] Kernan WN, Ovbiagele B, Black HR, Bravata DM, Chimowitz MI, Ezekowitz MD, et al Guidelines for the prevention of stroke in patients with stroke and transient ischemic attack: a guideline for healthcare professionals from the American Heart Association/American Stroke Association. Stroke; a journal of cerebral circulation 2014; 45(7): 2160–2236. 10.1161/STR.0000000000000024 24788967

