# Supplementary material for: Right-to-left shunt detection using contrast-enhanced transcranial Doppler: A comparison of provocation maneuvers between coughing and a modified Valsalva maneuver
Source: PLoS One. 2017 Apr 6;12(4):e0175049. doi: 10.1371/journal.pone.0175049 (PMC5383058; doi:10.1371/journal.pone.0175049)
Supplement: S1 File — (PDF) [file pone.0175049.s001.pdf]

The First Hospital of Jilin University

Ethics Committee Approval Form

Approved No. 2015-180

Title of research	The study of association between right-to-left shunt and migraine		
Study Source	The First Hospital of Jilin University		
Applicant Department	Neurology Department	Applicant	Prof. Ying-qi Xing
Review Class	Initial review	Review Type	Quick Review
Reviewers	Liu-ling Tan; Ming-li Rao.		
Approved Documents	1. Clinical study protocol.<br>2. Informed consent (written signature).<br>3. Case Report Form.<br>4. Resume of the lead researcher.		
Review comments	Approval		
Review Frequency	□ 3 Months □ 6 Months ■ 12 Months		
Effective Time	The effective time of the document is one year.		
Reviewers Signature	Liu-ling Tan		
Date	2015,05,08		

Ethics Committee of The First Hospital of Jilin University

吉林大学第一医院伦理委员会

临床试验与研究审批件

（2015 年）临审第（2015-180）号

试验项目名称	偏头痛与右向左分流相关性研究		
项目来源	吉林大学第一医院		
申请科室	吉林大学第一医院<br>神经内科	主要研究者	邢英琦
审查类别	初始审查	审查方式	快速审查
主审委员	谭毓铨；饶明俐		
批准文件	1.临床研究方案（版本号：1.0；版本日期：2015-4-25）<br>2.知情同意书（版本号：1.0；版本日期：2015-4-25）<br>3.调查登记表<br>4.主要研究者简历		
审查意见	同意		

根据卫生部《涉及人的生物医学研究伦理审查办法（试行）》（2007）、CFDA《药物临床试验质量管理规范（2003）》、《医疗器械临床试验规定（2004）》、WMA《赫尔辛基宣言》、CIOMS《人体生物医学研究国际道德指南》和《医疗器械监督管理条例》（2014）的伦理原则，经本伦理委员会审查，同意该临床研究在本中心开展。

请遵循 GCP 原则、遵循伦理委员会批准的方案开展临床研究，保护受试者的健康与权力。

研究过程中若变更主要研究者，对临床试验方案、知情同意书、招募材料等的任何修改，请申请人提交修正案审查申请。

发生严重不良事件，请申请人及时提交严重不良事件报告。

请申请人按照伦理委员会规定的年度/定期跟踪审查频率，在截止日期前 1 个月提交研究进展报告；当出现任何可能显著影响试验进行、或增加受试者危险的情况时，请申请人及时向伦理委员会提交书面报告。

研究纳入了不符合纳入标准或符合排除标准的受试者，符合中止试验规定而为未让受试者退出研究，给予错误治疗或剂量，给予方案禁止的合并用药等没有遵从方案开展的情况；或可能对受试者的权益/健康以及科学性造成不良影响等违背 GCP 原则的情况，请申办方/监察员/研究者提交违背方案报告。

申请人暂停/ 或提前终止临床研究，请及时提交暂停/终止研究报告。

完成临床研究，请申请人提交结题报告。

年度/定期跟踪审查频率	☐3 个月	☐6 个月	☑12 个月
有效期	本批件有效期为一年，须在截止日期前 1 个月递交研究进展报告，经伦理委员会批准后方可继续进行。		
(副)主任签字	谭毓铨		
日期	2015.5.8		

吉林大学第一医院伦理委员会
