# Supplementary material for: Right-to-left shunt detection using contrast-enhanced transcranial Doppler: A comparison of provocation maneuvers between coughing and a modified Valsalva maneuver
Source: PLoS One. 2017 Apr 6;12(4):e0175049. doi: 10.1371/journal.pone.0175049 (PMC5383058; doi:10.1371/journal.pone.0175049)
Supplement: S2 File — (PDF) [file pone.0175049.s002.pdf]

Informed Consent

We would like to invite you to participate in the study on the association between a right-to-left shunt and migraine, which has been approved by the ethics committee of the first hospital of Jilin University. In this study, 10 hospitals will jointly conduct the nationwide study. This study is estimated to include 1500–2000 volunteer subjects.

Why do we carry out this study?

Objective: The study intends to discuss the positive effect of a right-to-left shunt in patients with a migraine through this multi-centre, large sample study. In addition, we will analyse the positive rate of cerebral infarction without symptoms in patients with a migraine. Furthermore, we will analyse the association between different subtypes of a migraine and the right-to-left shunt. We hope to help the government and medical departments to develop a guide for migraine prevention and intervention. Moreover, we would like to provide an objective basis to guide the allocation of the medical resources.

How will the study be carried out?

The study intends to include patients with a migraine (about 1000 cases) who were diagnosed according to the criteria of a migraine, and healthy volunteers (about 500 cases). Healthy volunteers are age-matched subjects with no migraine. Exclusion criteria: severe stenosis of the artery, the temporal window through bad, no adequate cubital venous access, and inability to perform Valsalva manoeuvre because of severe heart or lung disease. All patients will undergo contrast-enhanced transcranial Doppler and transcranial Doppler. Magnetic resonance imaging is suggested for patients with a migraine.

What will I need to do in the study?

Please cooperate to record information carefully. The study will keep your personal information confidential. You may receive a follow-up call, and please cooperate seriously.

What benefit can I get from the study?

Participating in the study will not interfere with your normal diagnosis and treatment. The information obtained from the study will help doctors to analyse the causes of a migraine. We hope to discuss the association between the right-to-left shunt, migraine, and asymptomatic cerebral infarction.

Do I have to take part in the study?

The present study does not affect your normal examination. Participating in this study is voluntary. Participants can refuse to participate in the study and have the right to withdraw from the study at any time. Your decision will not influence your treatment. The fee for the examination is nominal.

The agreement statement of subjects

I have read the above information regarding this study and fully understand the present study. I am willing to participate in this clinical study.

Agree ☑ **Refuse** □

Signature: Ping Sun **Date:** 2016.01.08

Contact address: 18104312650

知情同意书

我们邀请您参加吉林大学第一医院伦理委员会批准开展的偏头痛与右向左分流相关性研究。本研究将在全国 10 家医院共同开展，估计将有 1500-2000 名受试者自愿参加。

为什么要开展本项研究？

研究目的：本研究拟通过多中心大样本量研究国人右向左分流阳性率，偏头痛患者右向左分流阳性率，偏头痛患者无症状脑梗死发生率，进一步分析不同亚型及偏头痛临床特征与右向左分流发生的相关性，梗死灶的分布特点，深入研究右向左分流、偏头痛和无症状脑梗死的关系。为政府和医疗主管部门制订偏头痛预防及干预指南及优化医疗资源配置提供客观依据。

该研究是怎样进行的？

研究分别纳入正常人群（约 500 例）与偏头痛患者（约 1000 例），其中偏头痛患者入组标准：严格根据偏头痛诊断标准明确诊断的患者。正常人群为年龄相匹配的非偏头痛患者，要求既往体健。排除标准：经颅多普勒超声证实存在严重颅内外大血管狭窄及闭塞的患者。对所有患者行发泡试验、心脏超声及经颅多普勒超声检查，并建议入组偏头痛患者行头 MRI。对采集信息进行统计分析。

研究中我该做什么又将受到何种影响？

请您认真配合记录相关信息，本研究将对您的个人信息进行保密，您可能接到随访电话，请认真积极配合。

从此研究中我能得到什么利益？

参加本研究不会对您的诊疗进行任何干预，但从本研究中得到的信息将有助于医生分析头痛的原因，深入探讨右向左分流、偏头痛和无症状脑梗死的关系。

我必须参加研究吗？

参加本研究完全不影响您的正常检查，正常收取检查费用，参加本研究是完全自愿的，您可以拒绝参加研究，或者研究过程中的任何时候选择退出研究，该决定不会影响您未来的诊治。

受试者同意声明：

我已经阅读了上述有关本研究的介绍，对参加本研究充分了解。我是自愿同意参加本文所介绍的临床研究。

我同意 ☑ 不同意 ☐

受试者签名：孙平　　　　日期：2016年01月08日

受试者联系电话：18104312650
