# Supplementary material for: Right-to-left shunt detection using contrast-enhanced transcranial Doppler: A comparison of provocation maneuvers between coughing and a modified Valsalva maneuver
Source: PLoS One. 2017 Apr 6;12(4):e0175049. doi: 10.1371/journal.pone.0175049 (PMC5383058; doi:10.1371/journal.pone.0175049)
Supplement: S3 File — (PDF) [file pone.0175049.s003.pdf]

CERTIFICATE OF ENGLISH EDITING

This document certifies that the paper listed below has been edited to ensure that the language is clear and free of errors. The logical presentation of ideas and the structure of the paper were also checked during the editing process. The edit was performed by professional editors at Editage, a division of Cactus Communications. The intent of the author's message was not altered in any way during the editing process. The quality of the edit has been guaranteed, with the assumption that our suggested changes have been accepted and have not been further altered without the knowledge of our editors.

Title Of The Paper

Right-to-left shunt detected detection using contrast-enhanced transcranial Doppler: A comparison of provocation maneuvers between coughing and a modified Valsalva maneuver

Authors

Sibo Wang

Job Code

XIQIN_24_3

Signature

Vikas Narang

Vikas Narang,
Vice President, Author Services, Editage

Date of Issue
February 24, 2017

Editage, a brand of Cactus Communications, offers professional English language editing and publication support services to authors engaged in over 500 areas of research. Through its community of experienced editors, which includes doctors, engineers, published scientists, and researchers with peer review experience, Editage has successfully helped authors get published in internationally reputed journals. Authors who work with Editage are guaranteed excellent language quality and timely delivery.

Contact Editage

Worldwide	Japan	Korea	China	Brazil	Taiwan
request@editage.com	submissions@editage.com	submit-korea@editage.com	fabiao@editage.cn	inquiry.brazil@editage.com	submitjobs@editage.com
+1 877-334-8243	+81 03-6868-3348	1544-9241	400-005-6055	0800-892-20-97	02 2657 0306
www.editage.com	www.editage.jp	www.editage.co.kr	www.editage.cn	www.editage.com.br	www.editage.com.tw
